# Evaluation of humoral and cellular immune responses in healthcare workers with varying levels of SARS-CoV-2 exposure: effects of CoronaVac vaccination followed by heterologous booster

**DOI:** 10.3389/fimmu.2025.1576430

**Published:** 2025-05-08

**Authors:** Shayane Martins Rodrigues Gomes, Marcelo Ribeiro-Alves, Roberto Stefan de Almeida Ribeiro, Andréia Carolinne de Souza Brito, Vinicius da Cunha Lisboa, Samara Galdino de Azevedo, Jeane de Souza Nogueira, Leda dos Reis Castilho, Luís Cristóvão Sobrino Pôrto, Silvia Amaral Gonçalves da Silva, Patrícia Maria Lourenço Dutra, Wânia Ferraz Pereira Manfro, Luciana Silva Rodrigues

**Affiliations:** ^1^ Discipline of Parasitology, Department of Microbiology, Immunology and Parasitology, Medical Science Faculty, Rio de Janeiro State University (UERJ), Rio de Janeiro, Brazil; ^2^ Laboratory of Clinical Research on ISTs/AIDS, National Institute of Infectology Evandro Chagas, Oswaldo Cruz Foundation (FIOCRUZ), Rio de Janeiro, Brazil; ^3^ Laboratory of Immunopathology (LIP), Discipline of General Pathology, Medical Science Faculty, UERJ, Rio de Janeiro, Brazil; ^4^ Laboratory of Histocompatibility and Cryopreservation, Tissue Repair and Histocompatibility Technologic Core, Rio de Janeiro State University, Rio de Janeiro, Brazil; ^5^ Cell Culture Engineering Lab, Chemical Engineering Program, Coordination of Graduate Engineering Programs, Federal University of Rio de Janeiro (UFRJ), Rio de Janeiro, Brazil; ^6^ Discipline of Microbiology and Immunology, Department of Microbiology, Immunology and Parasitology, Medical Science Faculty, UERJ, Rio de Janeiro, Brazil

**Keywords:** CoronaVac, humoral immunity, cytokines, heterologous booster, healthcare workers

## Abstract

**Background:**

The COVID-19 pandemic demanded diverse vaccination strategies, and there is significant interest in their effectiveness in generating a robust immune response. In Brazil, the use of CoronaVac was crucial in reducing mortality; however, heterologous booster doses were necessary to enhance memory immune response. This study aimed to evaluate the humoral and cellular immunity in healthcare workers who were vaccinated with a complete regimen of CoronaVac and subsequently received heterologous booster doses over nearly one year.

**Methods:**

A longitudinal study recruited healthcare professionals with varying levels of exposure to SARS-CoV-2 from the Health Complex of the Rio de Janeiro State University (UERJ), Rio de Janeiro, Brazil. Blood samples were collected at five time points, including baseline and after vaccination with CoronaVac and heterologous booster doses (ChAdOx1 nCov-19 or BNT162b2). The Th1/Th2/Th17 cytokine production was measured by Flow Cytometry, using whole blood samples stimulated or not with the SARS-CoV-2 Spike protein. In parallel, serum levels of IgG antibodies against Spike (anti-S) and Nucleocapsid (anti-N) proteins were assessed using an immunoassay. Adjustments were made for confounding factors, including age, sex, level of SARS-CoV-2 exposure, and COVID-19 infection status.

**Results:**

Our results demonstrate that CoronaVac induced high anti-S IgG levels at all evaluated time points (P<0.01). Cytokine analysis revealed a sustained production of antigen-specific Th1 cytokines, including IL-2 (P<0.01) and IFN-γ (P<0.05) regardless of level of SARS-CoV-2 exposure or previous COVID-19 infection at any point during the study. Additionally, we identified six moderate to strong positive correlations (P<0.0001): IL-10 and IFN-γ (ρ=0.77), IL-6 and TNF (ρ=0.77), IL-2 and IFN-γ (ρ=0.71), IL-6 and IL-10 (ρ=0.66), anti-N IgG and anti-S IgG (ρ=0.62), and IL-2 and anti-S IgG (ρ=0.62).

**Conclusion:**

The CoronaVac elicited an antigen-specific cellular immune response, characterized by enhancing the production of key cytokines such as IFN-γ and IL-2, with high levels of anti-S IgG. Furthermore, the administration of heterologous boosters significantly enhanced these immune responses, demonstrating induced-specific immunological response. These findings underscore the importance of primary vaccination and boosters in inducing immune protection against COVID-19, potentially informing future vaccination policies and approaches.

## 1 Introduction

The Coronavirus Disease 2019 (COVID-19) has posed a significant global health crisis, with over 7 million deaths reported worldwide by April 2025 (1). Vaccination emerged as a crucial strategy, employing various platforms to mitigate the SARS-CoV-2 infection. It helped manage disease severity and reduced mortality and morbidity rates, promoting health equity and economic growth (2, 3). In the face of the COVID-19 pandemic, the World Health Organization (WHO) and the Brazilian National Immunization Program (PNI) have initially prioritized the immunization of high-risk groups, such as the elderly, individuals with chronic diseases, healthcare professionals, and other vulnerable populations (4, 5).

In Brazil, the national vaccination campaign started with two primary vaccines: initially CoronaVac, an inactivated virus vaccine, developed by Sinovac Life Sciences (Beijing, China) in partnership with Butantan Institute/São Paulo, Brazil, followed by ChAdOx1 nCov-19 (AstraZeneca/University of Oxford) (6). The CoronaVac vaccination regimen involved two doses administered 28 days apart, with studies demonstrating significant efficacy, safety, and tolerability (7, 8). Additionally, heterologous boosters, including vaccines such as ChAdOx1 nCov-19 or BNT162b2 (Pfizer-BioNTech), were introduced to enhance immune response. Large-scale studies, including one conducted in Chile, have shown that CoronaVac is over 60% effective in preventing COVID-19 and more than 80% in reducing hospitalizations and fatalities (9). However, evidence shows that immunity declines over time, making booster doses necessary for its maintenance (10).

Protective and long-lasting immunity against COVID-19 depends on both humoral and cellular immune responses, which together constitute what is known as adaptive immunity. Humoral immunity is characterized by the production of antibodies and the generation of memory B cells, whereas cellular immunity involves the activation of CD4^+^ and CD8^+^ T cells. These T cells play crucial roles in orchestrating the immune response through the secretion of various cytokines (11). Cytokines are key signaling molecules that regulate and activate other immune cells, thereby modulating the inflammatory response and promoting pathogen elimination. Cytokines, secreted by immune cells such as B cells, T cells, dendritic cells, and macrophages, act on a range of target cells, including immune, endothelial, and epithelial cells (12). For instance, CD4^+^ T cells produce cytokines such as IL-2, IL-4, and interferon-gamma (IFN-γ), which are essential for B cell activation, the promotion of cytotoxic CD8^+^ T cell responses, and macrophage activation (13). Tanriover et al. (8) highlighted the importance of T cell responses in sustaining immunity, while Xu et al. (14) emphasized that the durability of these cellular responses is crucial for long-term protection against severe disease. Numerous studies have documented antibody production in the initial months following vaccination, particularly focusing on neutralizing antibodies as indicators of immediate protection. However, further investigation is needed to fully understand the specific contributions of both humoral and cellular immunity in the context of CoronaVac vaccination.

A previous study assessed the cellular immune responses to CoronaVac for up to a year, with findings revealing that the vaccine effectively induces long-term, antigen-specific CD4^+^ T cell responses in peripheral blood mononuclear cells (PBMC) upon stimulation with SARS-CoV-2 peptide megapools. In parallel, a balanced Th1- and Th2-type cytokine production was observed in supernatants from PBMC cultures (15). We recently reported a study employing flow cytometry to quantify antigen-specific cytokine profiles in a cohort of COVID-19 patients using whole blood stimulated with the Spike protein (16). That study revealed an enriched antigen-specific Th1-type cytokine profile in recovered COVID-19 patients, whereas individuals with Long-COVID exhibited significantly elevated levels of inflammatory cytokines IL-1β and IL-6 compared to the unexposed group, two months after acute infection. Building on this foundation, we now extend our investigation to the cellular immune responses induced by CoronaVac in a vaccinated cohort.

This longitudinal study investigated humoral (serology) and cellular (antigen-specific cytokine release assay, CRA) immune responses in healthcare workers with varying levels of exposure to SARS-CoV-2, classified according to WHO guidelines (17). Participants received the vaccine CoronaVac, followed by heterologous booster doses. Cellular immunity was assessed by profiling Th1-, Th2-, and Th17-type cytokines at multiple time points, using whole blood stimulated with recombinant SARS-CoV-2 Spike protein. Additionally, IgG levels against the Spike (anti-S) and Nucleocapsid (anti-N) proteins of SARS-CoV-2 were quantified. To our knowledge, this is the first study to longitudinally and integratively examine IgG responses to both structural viral proteins both alongside specific-antigen cytokine profiles in a cohort of CoronaVac-vaccinated healthcare workers in Brazil. This comprehensive approach enhances our understanding of the durability and magnitude of vaccine-induced immune response in a high-risk population under real-world conditions.

## 2 Materials and methods

### 2.1 Study design and participants

This longitudinal study included 61 healthcare workers aged 18 years or older, recruited from the Health Complex of the Rio de Janeiro State University (UERJ) in Brazil. Participants were categorized on their different levels of exposure to SARS-CoV-2 (17). Those working remotely without contact with infected individuals were classified as low risk; those in frequent close contact with individuals from areas with known or suspected transmission were classified as medium risk; those involved in patient screening and handling respiratory specimens as high risk; and those regularly exposed to aerosols containing SARS-CoV-2 were classified as very high risk.

**Figure 1:**
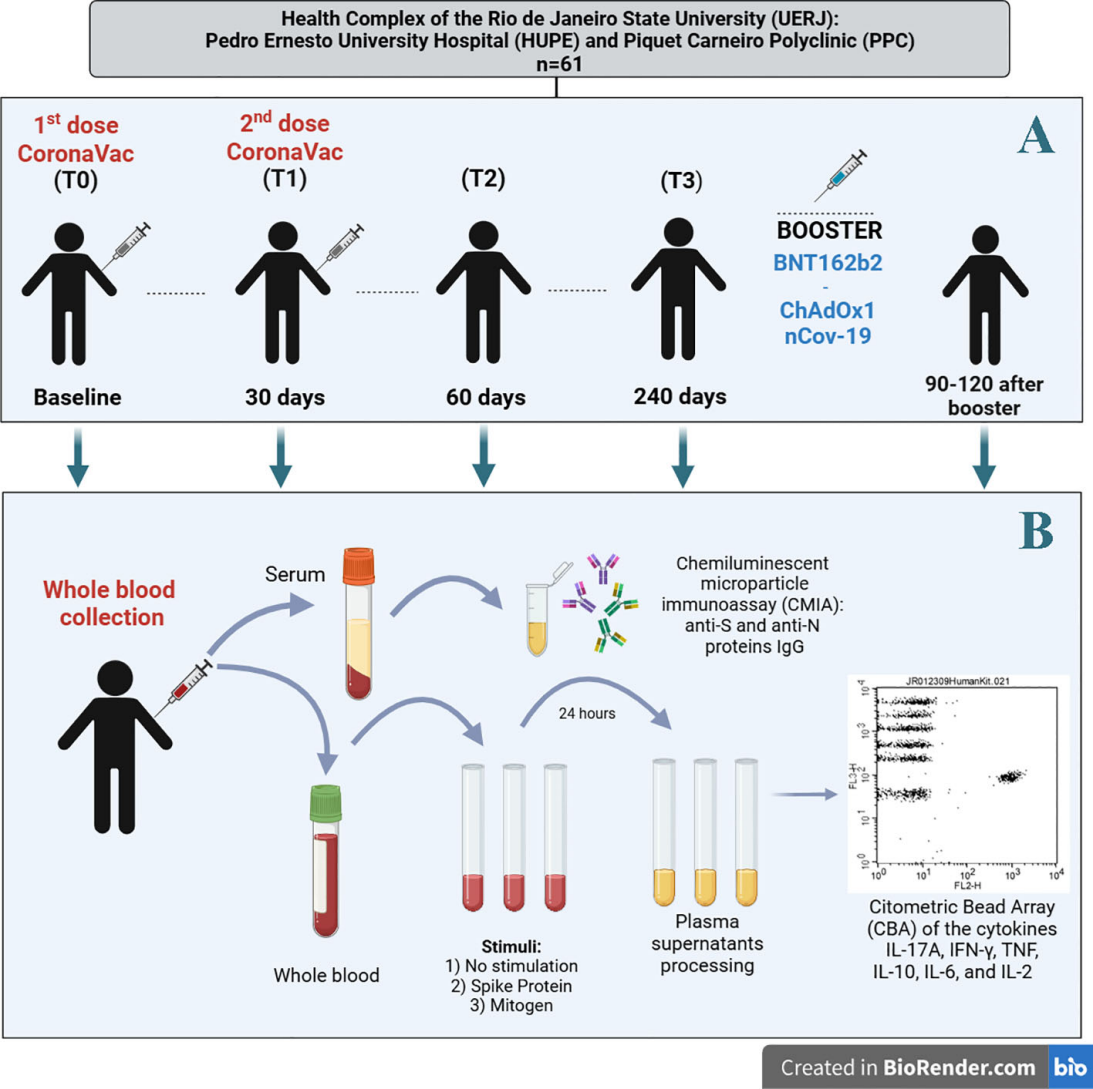
**(A)** Study design, **(B)** sample processing (serum and plasma) and immunoassays. (Figure created with the free version of BioRender). S (spike) and N (nucleocapsid) proteins. For whole blood and serum samples, the number of participants was as follows: T0 (N=61), T1 (N=61), T2 (N=61), T3 (N=35 for whole blood and N=61 for serum), and for the heterologous booster (N=14 for whole blood and N=61 for serum).

Regardless of prior SARS-CoV-2 infection status, all participants received the first and second doses of the CoronaVac vaccine between March and April 2021, adhering to the 28–30-day interval recommended by the Ministry of Health guidelines (18). Social and demographic data, health status, and COVID-19 monitoring information were self-reported via an electronic questionnaire. Participants were monitored until March 2022, during which peripheral blood samples were collected at the following time points for the whole blood anti-S protein stimulation assay (**Figure 1A**):

- **T0 (baseline):** At the administration of the first dose of CoronaVac.
- **T1 (second dose):** 28–30 days after the first dose.
- **T2:** 60 days after the first dose.
- **T3:** approximately 240 days after the first dose of CoronaVac;
- **Heterologous booster:** 90–120 days after receiving a heterologous booster vaccine, (ChAdOx1 nCoV-19 [Oxford-AstraZeneca] or BNT162b2 [Pfizer BioNTech]).

Eligibility for continued participation in the study analysis required the completion of at least three blood collections, with the baseline collection at T0 being mandatory.

### 2.2 Recombinant SARS-CoV-2 spike protein

To stimulate antigen-specific Tcells, recombinant SARS-CoV-2 Spike protein was produced in HEK293 cells, following a previously established protocol (19). The protein was expressed as a stabilized trimer in the pre-fusion conformation and purified from cell culture supernatants using affinity chromatography to ensure high purity. Endotoxin levels were quantified using the Pierce Chromatography Endotoxin Quant Kit (Thermo Fisher Scientific) and confirmed not to exceed 0.0052 EU/μg of protein.

For use in the cytokine release assay (CRA), the purified Spike protein was filtered and diluted in sterile endotoxin-free phosphate-buffered saline (PBS) before being added to the corresponding tubes.

### 2.3 Cytokine release assay

Peripheral blood samples were collected at T0, T1, T2, T3, and after the heterologous booster using vacuum tubes containing sodium heparin (BD Vacutainer, Becton Drive, Franklin Lakes, USA). One milliliter of whole blood was then transferred into sterile round-bottom polystyrene test tubes (Corning Science Mexico S.A. de C.V., Reynosa, Mexico). Samples were processed under the following conditions, as previously described by Gomes et al. (16): i) Negative control: no stimulation; ii) Recombinant Spike protein stimulation: 5 μg/mL; and iii) Positive control: mitogen stimulation at 5 μg/mL (phytohemagglutinin; Roche Diagnostics GmbH, Mannheim, Germany). Incubation was carried out for 24 hours at 37°C in a humidified atmosphere (5% CO_2_). Following incubation, samples were centrifuged at 3,000 g for 15 minutes at 24°C to collect plasma supernatant. Plasma samples were collected and stored at -80°C until cytokine measurements.

The concentrations of IFN-γ, TNF, IL-10, IL-6, IL-2, and IL-17A, were quantified in plasma supernatant samples obtained from cytokine release assay (each of three stimulation tubes) at T0, T1, T2, T3, and after the heterologous booster. Cytokine levels were measured using the Human Th1/Th2/Th17 Cytokine Kit (BD Bioscience, San Jose, CA, USA), based on multiplex cytometric bead array (CBA) technology, following the manufacturer’s protocol (**Figure 1B**). Data acquisition was performed using a BD FACSCanto™ II flow cytometer, and cytokine concentrations were expressed in pg/mL. Standard curves for cytokine quantification were generated within a range of 0 to 5,000 pg/mL. Antigen-specific cytokine responses were determined by subtracting the value from the negative control tube from those obtained in the recombinant Spike protein-stimulated tube.

### 2.4 Measurement of IgG antibodies against SARS-CoV-2 spike and nucleocapsid proteins

For serum collection, peripheral blood was drawn into vacuum tubes without anticoagulants, containing a clot activator (BD Vacutainer, San Jose, CA, USA) at T0, T1, T2, T3, and after the heterologous booster. The samples were left to rest at room temperature for at least 30 minutes to allow clotting. Subsequently, the samples were centrifuged at 815 g for 10 minutes at 25°C. Serum samples were collected and stored at -80°C until serology analysis (**Figure 1B**).

Serum samples were analyzed at the COVID-19 Diagnostic Support Unit (UNADIG) - Fiocruz/RJ diagnostic center for both qualitative and quantitative detection of IgG antibodies targeting the SARS-CoV-2 Nucleoprotein (anti-N) or Spike (anti-S) proteins. The antibody assessment was performed using automated immunoassays: SARS-CoV-2 IgG and SARS-CoV-2 IgG II Quant assays (Architect, Abbott – Illinois, USA). These assays utilize paramagnetic microparticles coated with either nucleoprotein or the receptor-binding domain (RBD) of the Spike protein’s S1 subunit. The assays are based on chemiluminescent microparticle immunoassay (CMIA) technology, and the tests were carried out following the manufacturer’s instructions. Results were reported as the Log10 of an index (signal/cut-off, S/C) for anti-N or Log10 in arbitrary units per milliliter (AU/mL) for anti-S. The positive thresholds for detection were defined as >1.4 for anti-N and >50.0 AU/mL for Anti-S.

### 2.5 Statistical analysis

To describe the sociodemographic and clinical characteristics of the study population, composed of healthcare professionals with different levels of exposure to SARS-CoV-2, appropriate statistical tests were applied. The Kruskal-Wallis tests were used for continuous numerical variables, while comparisons of the relative frequencies across different levels of nominal/categorical variables were performed using X2 tests. Besides, Pearson’s correlation coefficient was estimated for continuous numerical variables. In comparing the levels of Log10-transformed expression levels of cytokine S protein-specific production, IgG anti-S, and IgG anti-N (based on index values) across different vaccination time points with CoronaVac and a heterologous booster, the expected marginal mean values were obtained through mixed-effect multiple linear (Log-linear) regression models, including the main group effects, and the confounding variables age, sex, level of exposure to SARS-CoV-2, and active COVID-19 infection status in the fixed effects component of the models, and the participants identification as the random effect component of the models. Cumulative cytokine production (Log10-transformed) among groups with varying levels of exposure to SARS-CoV-2 was estimated by the cumulative integral with respect to time after T0 using trapezoidal integration. In comparing the levels of Log_10_. Cumulative cytokine production among levels of exposure to SARS-CoV-2, the expected marginal mean values were obtained through fixed-effect multiple linear (Log-linear) regression models, including the main group effects, and the confounding variables age, sex, and active COVID-19 infection status in the systematic component of the models. For both mixed- and fixed-effect multiple linear (Log-linear) regression models, marginal mean values and their 95% confidence intervals were then estimated by keeping all confounders in the multiple linear models at their mean values or equal proportions. Contrasts were constructed from these estimated marginal mean values. Pairwise p-values were corrected for the number of comparisons using the Holm-Sidak method. For the adjusted models, graphical analyses of the residuals were performed to confirm their randomness. A P-value ≤ 0.05 was used as the significance level in the analysis. All analyses were performed using R software version 4.1.2 (20), and packages ‘base’ for descriptive and correlation analyses, ‘pracma’ for trapezoidal integration, ‘lme4’ and ‘emmeans’ for model inferences, and their dependencies

## 3 Results

### 3.1 Demographic data of the study cohort

A total of 61 healthcare professionals were recruited for the study between March 2021 and March 2022 at T0, T1, and T2. The cohort had an average age of 46 years old (22-59), and 77% (47/61) of participants were female (**Table 1**). Blood samples for the assessment of cellular immunity, based on antigen-specific cytokine release assay (CRA), were also collected at T3 and 90–120 days after the heterologous booster from 35 and 14 individuals, respectively, due to variations in participant availability and adherence to the follow-up schedule.

**Table 1:** Characteristics of participants in the cohort.

Characteristics	N (%)
**Age, years** 22-59	61 (100)
**Gender** Female	47 (77)
**Level of exposure** Low Medium High Very high	6 (9.8) 11 (18) 9 (14.8) 35 (57.4)
**Comorbidities** Yes Hypertension Asthma Smoker Diabetes Obesity COPD/Emphysema	15 (24.60) 08 (13.11) 04 (6.56) 03 (4.92) 03 (4.92) 02 (3.28) 01 (1.64)
**COVID-19** Yes	28^a^ (45.9)
**Vaccine (T0, T1, T2, T3)** CoronaVac	61 (100)
**Vaccine (Booster)** BNT162b2 ChAdOx1 nCoV-19 N.I.	56 (91.80) 02 (3.3) 03 (4.9)

a Only one individual contracted COVID-19 during the blood collection. COPD, Chronic Obstructive Pulmonary Disease; N.I,: Not informed.

Regarding COVID-19 exposure, 57.4% (35/61) reported very high exposure, 14.8% (9/61) reported high exposure, 18.0% (11/61) had a medium exposure, and 9.8% (6/61) reported low exposure. Additionally, 24.6% (15/61) of participants reported one or more comorbidities. The most common comorbidities were hypertension (8/61), asthma (4/61), smoking (3/61), diabetes (3/61), obesity (2/61), and chronic obstructive pulmonary disease (COPD)/emphysema (1/61). A prior COVID-19 infection was reported in 44.2% (27/61) of individuals, and only one participant contracted COVID-19 during the study period.

All participants received CoronaVac for their primary and secondary vaccination doses. For the booster dose, 91.8% (56/61) received the BNT162b2 vaccine (Pfizer BioNTech), 3.3% (2/61) received the ChAdOx1 nCoV-19 vaccine (Oxford-AstraZeneca), and 4.9% (3/61) did not provide information about their booster dose.

In the subsequent sections, analyses of humoral and cellular immune responses were adjusted to account for potential biases related to exposure status and prior COVID-19 infection, ensuring accurate interpretation of the data.

### 3.2 Serological IgG responses against to SARS-CoV-2 proteins

**Figure 2:**
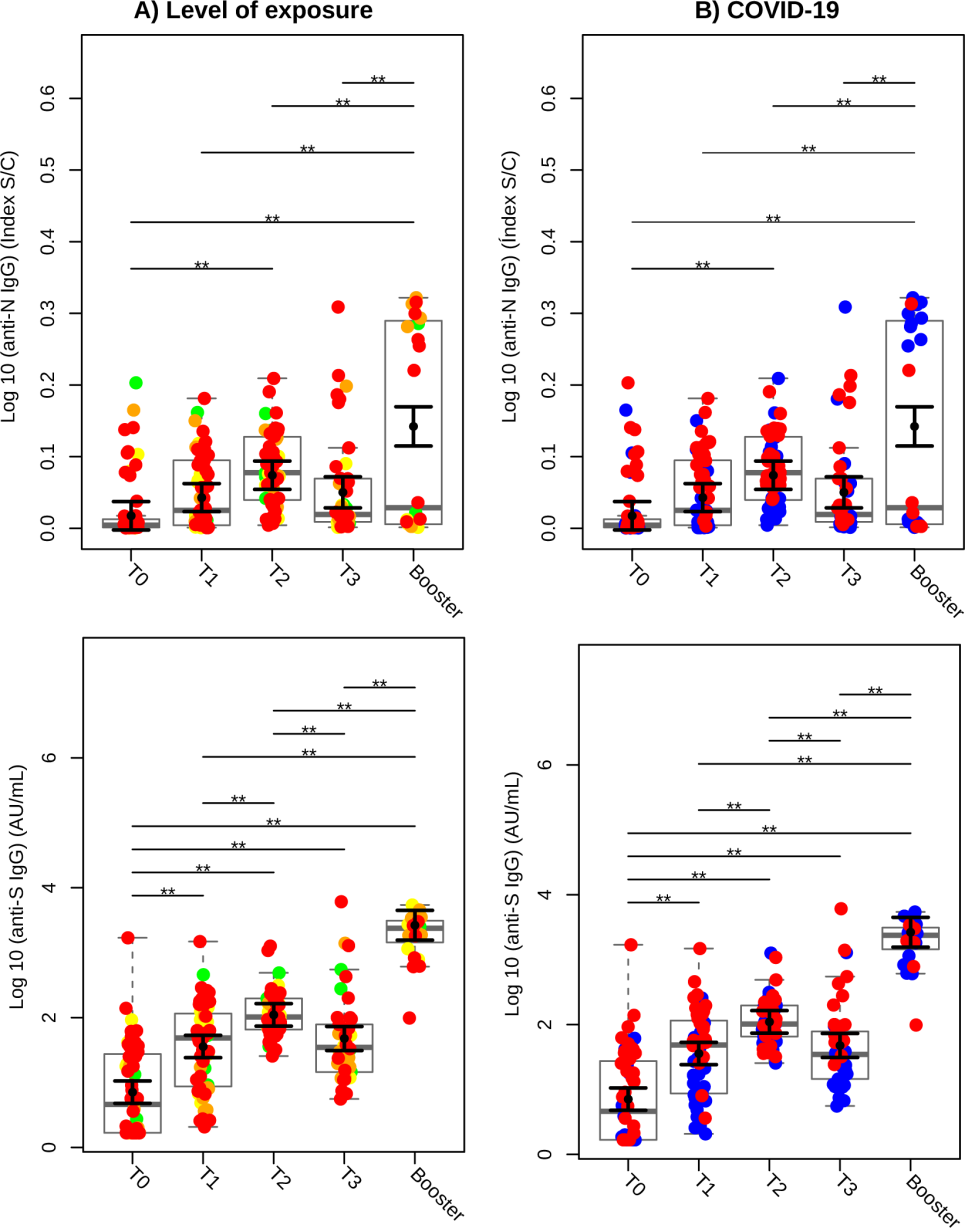
Serology of IgG against SARS-CoV-2 Nucleocapsid (anti-N) and Spike (anti-S) proteins over time. **(A)** Colors based on the level of exposure to SARS-CoV-2. Green circles represent individuals categorized as low; yellow, moderate; orange, high; and red, very high exposure levels. **(B)** Colors based on prior or subsequent infection with SARS-CoV-2. Blue circles indicate individuals who did not have COVID-19 before or during the study (COVID-19-negative), while red circles indicate individuals who had COVID-19 prior to or during the study (COVID-19-positive). Sampled serological levels obtained were analyzed in Log_10_ scale and illustrated using gray box and strip plots to compare the different blood collection time points. Each circle represents an individual, and the gray box plots show the interquartile range with the sample median represented by a solid gray centerline. The solid black circle and black vertical bars represent the adjusted means estimated by the linear mixed model and its 95% confidence intervals (CI), respectively. Marginal means values and their 95% CI were calculated across different collection time points, controlling for age, sex, exposure level, and SARS-CoV-2 infection (fixed effects) conditional to each participant (random effect). **, P<0.01.

**Figure 2A** illustrates the SARS-CoV-2 exposure levels of participants in our cohort, color-coded as follows: green for low exposure, yellow for moderate exposure, orange for high exposure, and red for very high exposure. A significant increase in IgG antibodies against SARS-CoV-2 nucleocapsid protein (anti-N) was observed at T2 compared to T0 (baseline), with a rise of 13.90% (P < 0.01). This increase was further amplified following the booster dose, with anti-N IgG levels rising by 33.26% compared to T0, 25.70% compared to T1, 16.99% compared to T2, and 23.60% compared to T3 (all with P < 0.01). At T0, 18.6% of participants tested positive for anti-N IgG, increasing to 46.7% at T1. At T2, 73.6% of participants tested positive for anti-N IgG, followed by a decline to 35.7% at T3 and 45.8% after the heterologous booster (P < 0.001).

IgG levels against the spike protein (anti-S) also showed significant increases at all measured time points compared to baseline (T0). At T1, anti-S levels increased 4-fold, at T2 they rose 14.5-fold, and at T3, they increased 5.6-fold (P < 0.01). Following the booster dose, anti-S IgG levels were significantly higher than at earlier time points (P < 0.01). A marked increase in anti-S IgG levels was observed at T2 compared to T1 (P < 0.01), although a significant decline was noted at T3 compared to T2 (P < 0.01).

**Figure 2B** stratifies antibody responses based on participants´ COVID-19 infection history. Individuals with no prior or concurrent COVID-19 infection are represented in blue, while those with a history of infection are shown in red. Curiously, during the heterologous booster phase, seven individuals in COVID-19-negative group (blue) exhibited elevated IgG production against nucleocapsid protein.

### 3.3 Antigen-specific cytokine response profile in individuals vaccinated with CoronaVac and a heterologous booster

Cytokines secreted by various cells play a crucial role in the polarization of CD4 T cells into distinct effector phenotypes, which, in turn, reinforce their identity through the secretion of signature cytokines (21, 22). To assess the cellular immune response triggered by the CoronaVac vaccine and a heterologous booster, whole blood from volunteers was stimulated with the SARS-CoV-2 Spike protein, and Th1/Th2/Th17-type cytokines were measured in the supernatants of the cytokine release assay.

**Figure 3:**
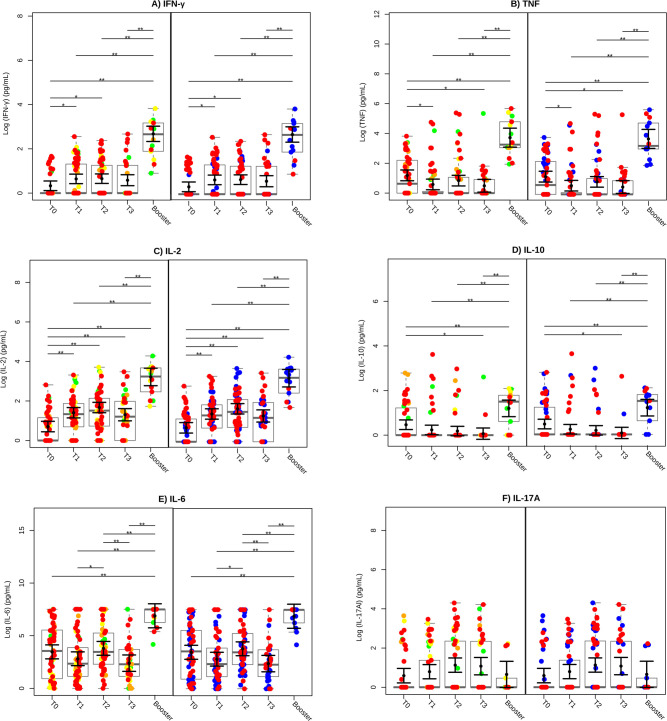
Cytokine production profile after vaccination. The cytokines IFN-γ **(A)**, TNF **(B)**, IL-2 **(C)**, IL-10 **(D)**, IL-6 **(E)**, and IL-17A **(F)** were evaluated in whole blood stimulated with SARS-CoV-2 Spike protein. Green circles represent low, yellow circles denote moderate, orange circles indicate high and red circles signify very high levels of exposure. SARS-CoV-2 infection status is also indicated, with blue circles representing individuals who did not have COVID-19 (COVID-19-negative) and red circles representing individuals who had COVID-19 (COVID-19-positive). SARS-CoV-2 Spike protein-specific cytokine levels were transformed to a Log_10_ scale and depicted using gray box and strip plots to compare the different blood collection time points. Each circle represents an individual, and the gray box plots show the interquartile range with the sample median represented by a solid gray centerline. The solid black circle and black vertical bars represent the adjusted means estimated by the linear mixed model and its 95% confidence intervals (CI), respectively. Marginal means values and their 95%CI were calculated across different collection time points, controlling for age, sex, exposure level, and SARS-CoV-2 infection (fixed effects) conditional to each participant (random effect). **, P<0.01.

A significant increase in IFN-γ production was observed at both T1 and T2 compared to baseline (T0) (P < 0.05), with a further elevation following the booster, surpassing all previous time points (P < 0.01) (**Figure 3A**). In contrast, TNF production decreased at T1 and T3 compared to T0 (P < 0.05), but increased significantly post-booster, reaching levels higher than those observed at any time point (P < 0.01) (**Figure 3B**). IL-2 levels demonstrated significant increases at T1, T2, and T3 compared to baseline (P < 0.01), with an additional rise following the booster, which exceeded all prior time points (P < 0.01) (**Figure 3C**). IL-10 levels decreased at T3 compared to T0 (P < 0.05), but significantly increased after the heterologous booster, reaching levels higher than at any prior time points (P < 0.01) (**Figure 3D**). IL-6 production increased at T2 compared to T1 (P < 0.05), declined at T3 compared to T2 (P < 0.01), and increased significantly following the booster compared to all other time points (P < 0.01) (**Figure 3E**). In contrast, IL-17A levels remained stable, with no significant changes across the different time points (**Figure 3F**). These results underscore the pivotal role of the second dose of CoronaVac in inducing a robust cellular immune response, with IFN-γ and IL-2 emerging as key cytokines. Following the heterologous booster, IL-10 became more prominent, suggesting a shift toward a balanced and regulated immune response.

To further assess the balance between pro-inflammatory and anti-inflammatory immune responses, we analyzed the ratios of IFN-γ/IL-10, TNF/IL-10, IL-2/IL-10, and IL-6/IL-10 (**Supplementary Figure S1**). The IFN-γ/IL-10 ratio increased significantly at T2 compared to T0 (P < 0.01), indicating a predominant pro-inflammatory immune response. While the TNF/IL-10 ratio showed no significant changes across time points, the IL-2/IL-10 ratio increased significantly at T1, T2, and T3 compared to baseline (P < 0.01). Similarly, the IL-6/IL-10 ratio rose significantly at T2 compared to T0 (P < 0.01), suggesting that the second dose of CoronaVac elicited a strong inflammatory response critical for immune protection.

The cumulative cytokine production across groups with varying SARS-CoV-2 exposure levels is shown in the graph, which was generated based on the area under the curve (AUC) calculated from the mean cytokine expression of each participant (**Supplementary Figure S2**). A trend toward higher cumulative cytokine concentration, particularly for IFN-γ (A), TNF (B), IL-2 (C), IL-10 (D), IL-6 (E), and IL-17A(F), was observed as exposure level increased. However, these differences were not statistically significant. To further examine the dynamics of antigen-specific cytokine production over the 345-days follow-up period, AUC graphs were generated for each cytokine and participant. Representative examples for each exposure level, including both COVID-19-positive and -negative individuals, are detailed in **Supplementary Figures S3**-**S6**. At varying exposure levels to SARS-CoV-2, a distinct dynamic in cytokine production was observed. At very high exposure level (**Supplementary Figure S3**), COVID-19-positive individuals demonstrated a continuous increase in IFN-γ, IL-2, IL-6, and IL-17A, while TNF peaked early and IL-10 showed minimal expression. A similar pattern was observed in COVID-19-negative individuals. Among participants with high exposure (**Supplementary Figure S4**), both COVID-19-positive and -negative individuals exhibited multiple peaks in cytokine production. At moderate exposure level (**Supplementary Figure S5**), the COVID-19-positive individual exhibited higher cytokine peaks following the heterologous booster, whereas the COVID-19-negative individuals showed attenuated responses for IFN-γ, TNF, and IL-10. At low exposure levels (**Supplementary Figure S6**), COVID-19-positive individuals maintained consistent production of TNF, IL-2, IL-10, and IL-6, with multiple IFN-γ peaks. In contrast, COVID-19 negative individuals showed lower-magnitude responses for TNF, IL-10, and IL-17A but exhibited multiple peaks in IL-2, IL-6, and IFN-γ production.

### 3.4 Correlation analysis among humoral and cellular immune responses

**Figure 4:**
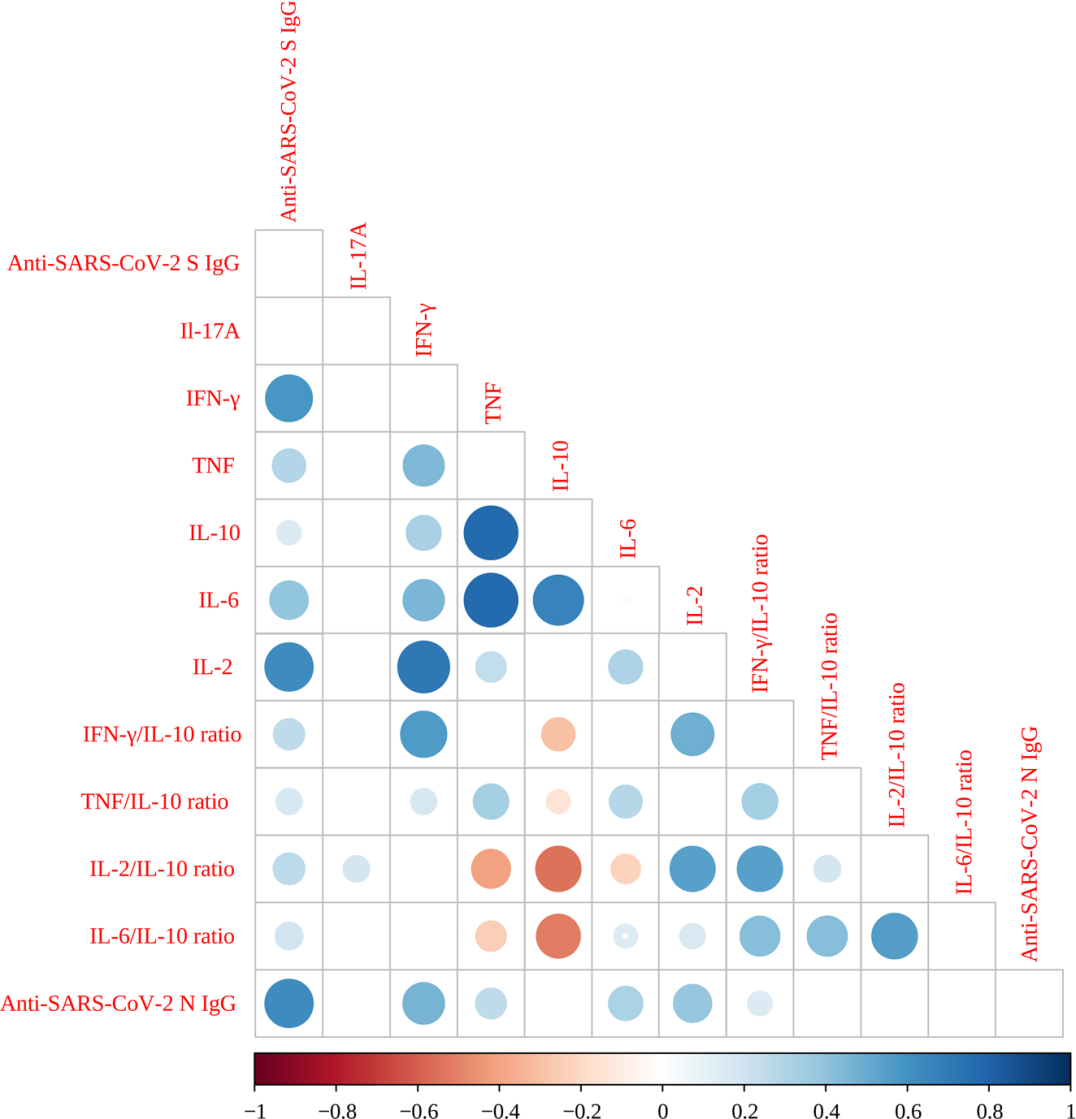
Correlogram of SARS-CoV-2 Spike protein-specific cytokine levels, cytokines ratios and serology. Very high positive (ρ=0.9-1.0) or high positive correlations (ρ=0.7-0.9) are indicated by large, dark blue circles. Moderate positive correlations (ρ=0.5-0.7) are indicated by medium-sized circles with a lighter blue color. Very high (ρ=0.9-1.0) or high negative correlations (ρ=-0.7 to -0.9) are indicated by large, red circles, while moderate negative correlations (ρ=-0.5 to -0.7) are shown by medium-sized circles with a lighter red color. Negligible correlations (ρ=0.0 to 0.3 or ρ=0.0 to -0.3) are indicated by small circles with pale (blue or red), while non-significant correlations as represented by empty squares. All Pearson correlations were adjusted by age, sex, exposure level, comorbidities, and SARS-CoV-2 infection status.

Adjusted Pearson correlation coefficients (ρ) were calculated to evaluate the relationships among cytokines, cytokine ratios, and serological markers (**Figure 4**). Six moderate-to-strong correlations were identified: IL-10 and TNF ρ=0.77 (P < 0.0001), IL-6 and TNF ρ=0.77 (P < 0.0001), IL-2 and IFN-γ ρ=0.71 (P < 0.0001); IL-6 and IL-10ρ=0.66 (P < 0.0001); anti-N IgG and anti-S IgG ρ =0.62 (P < 0.0001), and; IL-2 and anti-S protein IgG ρ=0.62 (P < 0.0001). Additionally, six moderate correlations were observed: IFN-γ and anti-S IgG ρ=0.58 (P<0.0001), IFN-γ/IL-10 ratio and IFN-γ ρ=0.56 (P<0.0001), IL-2/IL-10 ratio and the IFN-γ/IL-10 ratio ρ=0.54 (P<0.0001), IL-2/IL-10 ratio and IL-2 ρ=0.54 (P<0.0001), IL-6/IL-10 ratio and IL-2/IL-10 ratio ρ=0.55 (P<0.0001), and IFN-γ/IL-10 ratio and IL-2 ρ=0.48 (P<0.0001). A comprehensive summary of all correlation data among the study variables is provided in **Supplementary Table 1**.

## 4 Discussion

The COVID-19 pandemic has presented unprecedented challenges to healthcare professionals and scientists worldwide, necessitating rapid and effective solutions to develop vaccines to mitigate its devastating impact. Both humoral and cellular immunity are essential components of the immune response against SARS-CoV-2 infection, each playing a distinct yet complementary role in protection. CoronaVac, an inactivated whole-virus vaccine, has been widely deployed globally and has demonstrated effectiveness in preventing severe COVID-19 cases (7, 9). However, the humoral and cellular immune response elicited by CoronaVac, particularly among healthcare professionals varying levels of exposure to SARS-CoV-2, remains incompletely characterized.

In this longitudinal study, we assessed the immune response of healthcare workers at the Rio de Janeiro State University (UERJ) Health Complex following primary vaccination with CoronaVac and subsequent heterologous booster doses. Approximately 70% of participants reported high or very high exposure to SARS-CoV-2, highlighting the substantial occupational risk faced by this cohort. Our findings demonstrated that IgG antibodies against the S protein were detectable after both the first and second doses, whereas IgG targeting the N protein emerged only following the second dose. Notably, seven participants in the COVID-negative group tested anti-N positive after the booster dose. In these particular cases, we hypothesize that these individuals may have acquired the infection asymptomatically, as the Omicron variant wave occurred between time point T3 and the heterologous booster. Together, these results emphasize the critical role of the second dose of CoronaVac in achieving the peak IgG levels prior to the booster dose. Furthermore, our data confirm that CoronaVac elicits a robust antibody response against the spike protein, as evidenced by the sustained anti-S IgG levels over time.

Furthermore, our study revealed a robust cellular immune response regardless of prior COVID-19 infection or exposure status, as evidenced by the significant production of Th1 cytokines, such as IFN-γ and IL-2 following both the initial and second vaccine doses, which further enhancement observed after the highlighting how exposure levels might shape heterologous boosters. Notably, as expected, heterologous boosters also induced a pronounced increase in IL-10 levels, suggesting an augmented regulatory immune response post-vaccination. The elevated IFN-γ/IL-10 ratio indicates a well-regulated balance between pro-inflammatory and anti-inflammatory responses, with a predominance of the Th1 response, contributing to effective cellular immunity. The sustained IL-2/IL-10 ratio suggests that CoronaVac not only initiated a strong cellular immune response but also maintained it over time, which is essential for long-lasting protection. The IL-6/IL-10 ratio further emphasizes the role of the second dose in triggering an intense inflammatory response, crucial for an effective antiviral immunity.

Recent studies have provided valuable insights into the cellular immune responses triggered by various COVID-19 vaccines. For example, mRNA vaccines such as Moderna’s mRNA-1273 and Pfizer-BioNtech’s BNT162b2 have demonstrated robust and durable responses, both in antibody production and T cell activation. These vaccines induce high levels of anti-spike antibodies and potent T cell responses, including the generation of memory T cells, which are critical for long-term immunity (23). Similarly, CoronaVac has been shown to elicit a substantial CD4^+^ T cell response, sustained for at least six months post-vaccination. This vaccine primarily targets spike protein from SARS-CoV-2 and promotes the activation of follicular helper T cells (Tfh), leading to a balanced Th1 and Th2 cytokine profile. Additionally, CoronaVac maintains a strong humoral response, with elevated IgG antibody levels persisting for up to one year (15). However, neither CoronaVac nor ChAdOx1-S, the vaccines investigated in our cohort, are currently part of Brazil’s vaccination program. This shift reflects a strategic transition to mRNA-based vaccines, which are aimed at addressing evolving epidemiological challenges and providing more durable immune protection.

The assessment of the immune response among healthcare professionals following COVID-19 vaccination is critical for understanding vaccine effectiveness and the level of protection afforded to these high-risk individuals. In our cohort, 54.1% of participants had contracted COVID-19 prior to vaccination and study enrollment. This elevated infection rate likely reflects both the relaxation of lockdown measures at the end of 2020 (24) and the ongoing exposure healthcare workers face. Previous studies have identified employment in healthcare settings as a key predictor of infection risk among unvaccinated individuals (25). Notably, only one participant experienced a breakthrough infection during the study period, suggesting that vaccination may elicit a protective immune response. Supporting evidence from other studies further underscores that IgG seroprevalence against SARS-CoV-2 is higher among healthcare professionals with direct contact with COVID-19-positive patients, particularly those working in intensive care units, where exposure risk is markedly increased (25, 26).

Regarding vaccine platforms, evidence suggests that healthcare professionals vaccinated with an adenovirus vector vaccine exhibit a higher seroconversion rate compared to those who received the inactivated virus vaccine, CoronaVac (27). Furthermore, Ortega et al. (28) found that healthcare workers with prior SARS-CoV-2 infection achieved 100% seroconversion following CoronaVac vaccination, whereas individuals without a history of COVID-19 infection did not exhibit the same level of seroconversion. These findings highlight that both the type of vaccine platform and previous exposure to the virus significantly influence the immune response among healthcare professionals. To account for exposure-related biases, we employed a statistical modeling approach in this study, as detailed in the Methods section. This method allowed us to analyze humoral and cellular immune responses independently of prior COVID-19 and occupational exposure status.

In our study, we observed that the CoronaVac vaccine induced a higher production of anti-spike protein IgG at T2 (60 days after the first dose or 30 days after the second dose). Although a decline in anti-spike protein IgG production was noted after this time point, a high and significantly sustained level of this antibody persisted until 240 days post-vaccination. These findings align with the study by Costa et al. (15), which reported sustained production of anti-spike protein IgG for up to 365 days following CoronaVac administration. Our results also support previous studies indicating that the second vaccine dose is critical for achieving peak anti-spike protein IgG levels (15, 27). Similarly, mRNA vaccines and ChAdOx1-S have been shown to elicit high levels of IgG against the RBD/S1 portion of the spike protein within a few weeks after the second dose (29). However, the immune response observed with these vaccines was more robust compared to CoronaVac (27). This difference can be partially attributed to the intensity of dendritic cells (DCs) activation. For mRNA vaccines, RNA sensors such as TLR7 and MDA5 are activated, while the ChAdOx1-S vaccine activates TLR9. This leads to the production of pro-inflammatory cytokines and type I interferons, which promote the differentiation of T cells into helper and cytotoxic T cells. Additionally, Tfh cells play a crucial role in the activation of Spike protein-specific B cells, promoting the production of high-affinity antibodies, contributing to a stronger and more durable immune response (30). Nonetheless, CoronaVac has also been shown to induce Tfh activation, as observed in another study (15), indicating that this pathway is not exclusive to mRNA and viral vector vaccines, although the magnitude and persistence of the response may differ.

Since CoronaVac is produced using an inactivated whole virus, the generation of anti-nucleocapsid-IgG antibodies is also expected. Furthermore, studies suggest that prior exposure to other endemic coronaviruses may elicit a pre-existing immune response through cross-reactivity (31, 32). In our study, at time T0 (baseline), three COVID-19 negative individuals (in blue) exhibited a positive response for anti-nucleocapsid antibodies, suggesting that these cases may represent asymptomatic infections, as described in the literature (33) or cross-reactivity with other coronaviruses (32). Notably, unlike anti-spike protein IgG, which remained at sustained levels over time, anti-nucleocapsid IgG production showed a significant increase only at T2 (60 days after the first dose), followed by a decline at T3 (240 days after the first dose), indicating lower persistence compared to anti-spike IgG. These findings align with the study by Costa et al. (15), which reported a significant increase in anti-nucleocapsid IgG production after two doses of CoronaVac, followed by a decline observed at 180 days. Additionally, we observed that the heterologous booster dose effectively enhanced anti-nucleocapsid IgG, surpassing those induced by CoronaVac alone. Consistent with our findings, Demirhindi et al. (34) demonstrated that a heterologous regimen consisting of a CoronaVac prime (two doses) followed by BNT162b2 (Comirnaty^®^)/Pfizer-Biotech booster elicited a stronger immune response than a homologous inactivated vaccine regimen. When analyzing the antibody response in relation to COVID-19 infection, we found that participants with prior COVID-19 exposure exhibited higher levels of both anti-nucleocapsid and anti-spike IgG antibodies, suggesting that prior infection amplifies the vaccine-induced immune response. This observation aligns with findings from a study of healthcare workers vaccinated with CoronaVac, which reported high rates of anti-nucleocapsid IgG antibody positivity and elevated anti-RBD IgG antibody levels among participants working in medium to high-risk areas for COVID-19 (35).

Numerous studies have demonstrated that administering a booster dose (third vaccine dose) significantly increases anti-spike protein IgG production (29, 36, 37). Consistently, our study showed a marked increase in anti-spike protein IgG levels following the booster dose compared to homologous vaccination with CoronaVac. Notably, in this study, most booster doses were administered with the Pfizer vaccine, with only three participants receiving the AstraZeneca vaccine. These findings further reinforce the efficacy of booster doses in strengthening the immune response, underscoring their crucial role in optimizing vaccine-induced immunity.

Neutralizing antibodies are widely recognized as key immunological correlates of protection against SARS-CoV-2 (38). Although our study did not directly assess neutralizing antibodies, previous research has shown that mRNA-1273 vaccination induces potent neutralizing antibodies against major SARS-CoV-2 variants (39). Regarding CoronaVac, a study reported that the vaccine elicited neutralizing antibodies in 70% of vaccinated individuals without prior SARS-CoV-2 infection and in 95% of those with a history of COVID-19 infection, highlighting its effectiveness in generating neutralizing responses (28). Zhao et al. (40) observed a decline in neutralizing antibodies three months after the second dose of CoronaVac. However, an increase was noted in the sixth month, followed by a decline at 12 months, although with levels still higher than baseline.

In our study, IL-2 production remained sustained for up to 240 days following immunization with CoronaVac. Additionally, antigen-specific IFN-γ production was elevated up to 30 days after the second dose, suggesting a Th1-skewed response, although the specific cell subpopulation producing these cytokines were not characterized. In contrast, TNF levels decreased over time, while IL-6 exhibited only a transient increase after the second dose, with no sustained elevation. These findings align with another study (15) that reported high levels of IL-2, IL-1β, IFN-γ, and TNF in response to a MegaPool of CD4+ T cells-targeted peptides derived from the spike protein. A study from Chile found that 28 days after the second CoronaVac dose, only IL-8 showed a significant increase. This study also reported a rise in IFN-γ spot-forming cells (SFCs) specifically responding to recombinant spike protein, as well as an increase in granzyme SFCs, indicative of CD4^+^ and CD8^+^ T cells activation, respectively (41). Additionally, another investigation showed that CoronaVac induces spike protein-specific CD4^+^ T cell responses lasting up to 180 days, whereas no significant CD8^+^ T cell responses were detected (15). Another study (40) demonstrated that memory CD4+ T cells specific to the RBD portion of SARS-CoV-2 increased in the first month following vaccination with CoronaVac, and declined only after six months. In contrast, memory effector CD8+ T cells were detected in the first month after vaccination, peaked in the third month, and gradually declined over time. These collective findings highlight the complex cytokine dynamics and T cell response elicited by CoronaVac, emphasizing its capacity to activate components of the adaptive immune system, particularly CD4^+^ T cells.

As expected, the booster dose significantly increased cytokine production, including IFN-γ, TNF, IL-2, IL-10, and IL-6, with the exception of IL-17A. However, mRNA vaccines have been shown to induce a more pronounced pro-inflammatory cytokine profile (42, 43). For instance, Benhamouda et al. (42) reported that one month after the second dose of the Pfizer vaccine, individuals with prior SARS-CoV-2 infection exhibited elevated levels of CD4^+^ T cell-derived cytokines, including IFN-γ, IP-10, and TNF. Additionally, IL-2, TNF, IL-9, and IP-10 were identified as predictive markers for antigen-specific CD8^+^ T cells frequencies, a finding corroborated by murine model experiments. Similarly, Rosati et al. (43) highlighted a distinct cytokine and chemokine signature with a stronger pro-inflammatory profile following mRNA vaccination, including increased levels of IL-15, IFN-γ, CXCL10/IP-10, IL-6, TNF, CCL4/MIP-1β, IL-1Ra, IL-10, and IL-27. These findings underscore the complexity of the immune response to SARS-CoV-2 vaccination and suggest that cytokine dynamics are shaped by prior exposure and vaccine platform.

In our study, strong correlations (ρ > 0.7) between IL-10 and TNF, IL-6 and TNF, and IL-2 and IFN-γ (all with P < 0.0001) indicate a robust interplay between pro-inflammatory cytokines, potentially reflecting the simultaneous activation of inflammatory pathways and the innate immune response after vaccination. The observed correlations between IL-6, IL-10, and IL-2 (ρ = 0.66 to 0.77) further suggest a complex regulatory balance between pro-inflammatory and immunomodulatory cytokines. Additionally, the correlation between anti-S and anti-N IgG antibodies (ρ = 0.62) reinforces the link between the humoral immune response and cytokine production. The moderate correlations between cytokine ratios (such as IFN-γ/IL-10 and IL-2/IL-10) with other cytokines and serological markers suggest that these ratios may serve as indicators of overall immune status, reflecting the dominance of Th1, Th2, or a more balanced immune response.

We acknowledge the limitations in the present study: i) the focus on healthcare professionals may limit the generalizability of our findings to the broader population, as this group experiences higher exposure levels and distinct occupational health factors; ii) the relatively small sample size; iii) the absence of direct assessment of neutralizing antibodies, which are crucial for evaluating vaccine-induced protection; iv) the lack of functional assays, preventing a comprehensive characterization of T cell subpopulations and innate cells, which share the capacity to produce some cytokines, such as TNF, IL-4, and IL-10; v) although we measured Th1/Th2/Th17 cytokines, the Th17 response was not extensively explored; and vi) while we accounted for prior COVID-19 infection status, its potential heterogeneous effects on immune response may not have been fully controlled for. Despite of these limitations, our study has several strengths: a) the longitudinal design enable the assessment of both the early sustained immune response following vaccination, providing valuable insights into the dynamics of immunity over time; b) correlation analyses revealed significant positive associations between cytokines (e.g., IL-2 and IFN-γ) and IgG levels, offering a deeper understanding of the interplay between humoral and cellular immunity; c) similar to previous work by our group (16), we employed SARS-CoV-2 antigen-specific cytokine measurement model using whole blood (cytokine release assay, CRA), which better reflects the dynamic interactions between innate and adaptive immunity compared to conventional peripheral blood mononuclear cells (PBMC) cultures, and d) by targeting a high-risk population – healthcare professionals – our findings reinforce the importance of protecting frontline workers and provide critical evidence to inform vaccination policies, particularly regarding the role of booster doses in enhancing immune responses.

Taken together, our findings provide robust evidence that CoronaVac elicits a strong adaptive immune response to SARS-CoV-2, characterized by increased Th1 cytokines, particularly IFN-γ and IL-2. This response likely contributes to protective immunity, effectively supporting viral clearance while sustaining humoral responses over time. Clinically, these findings underscore the crucial role of vaccination in safeguarding healthcare professionals, who remain at heightened risk of exposure. Notably, our results align with those of Tanriover et al. (8), who reported over 80% vaccine efficacy in preventing COVID-19, with healthcare professionals comprising the majority of the study cohort. These observations reinforce the critical role of vaccination in protecting frontline workers and mitigating the burden of SARS-CoV-2 infection.

## 5 Conclusion

Our findings demonstrate that CoronaVac, in combination with a heterologous booster, effectively stimulates both humoral and cellular immune responses, regardless of natural prior COVID-19 infection or degree of exposure. The second dose played a key role in optimizing antibody production and SARS-CoV-2 antigen-specific cytokine response, while the booster further strengthened IFN-γ and IL-2 levels, promoting a well-regulated immune balance. Correlation analyses highlighted strong links between humoral and cellular markers, emphasizing the integrated nature of vaccine-induced immunity. These insights reinforce the importance of evaluating both arms of the immune system to refine vaccination strategies and enhance long-term protection against COVID-19.

## Data Availability

The original contributions presented in the study are included in the article/**Supplementary Material**. Further inquiries can be directed to the corresponding author.
