# Supplementary figures and images for: Evaluation of humoral and cellular immune responses in healthcare workers with varying levels of SARS-CoV-2 exposure: effects of CoronaVac vaccination followed by heterologous booster
Source: Front Immunol. 2025 May 8;16:1576430. doi: 10.3389/fimmu.2025.1576430 (PMC12095306; doi:10.3389/fimmu.2025.1576430)

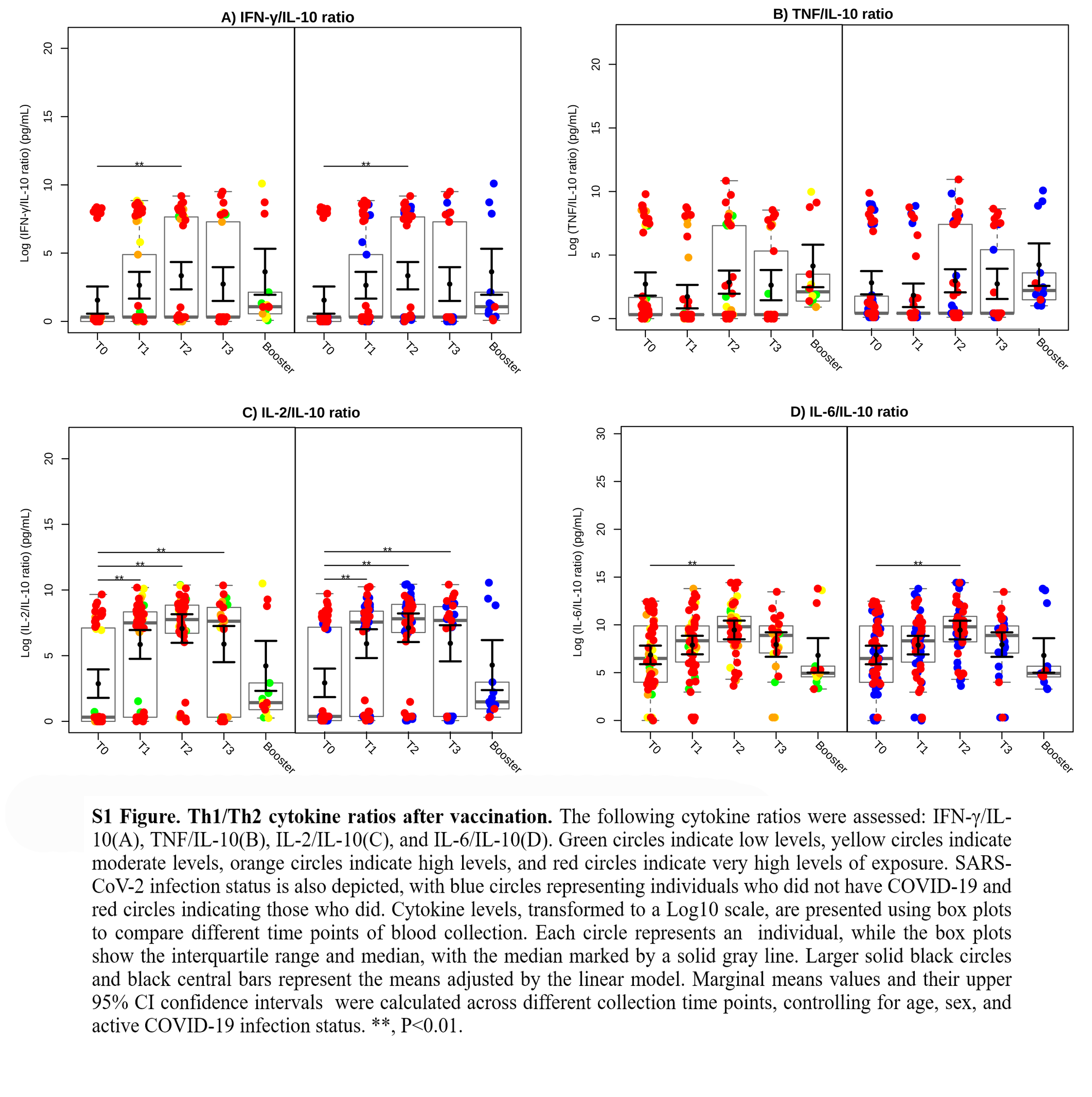

Supplement: Supplementary file 1 [file Image1.tif]

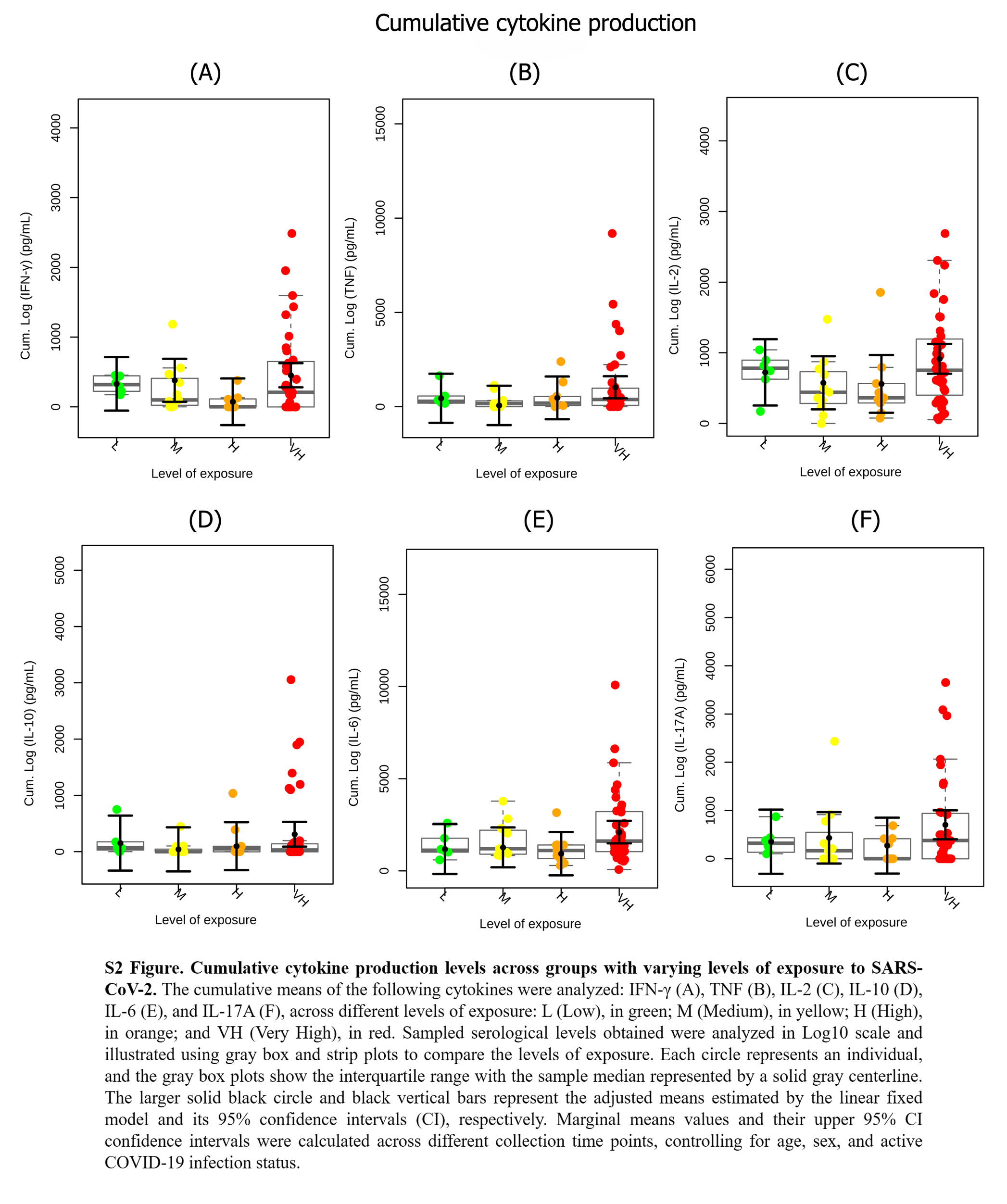

Supplement: Supplementary file 2 [file Image2.tif]

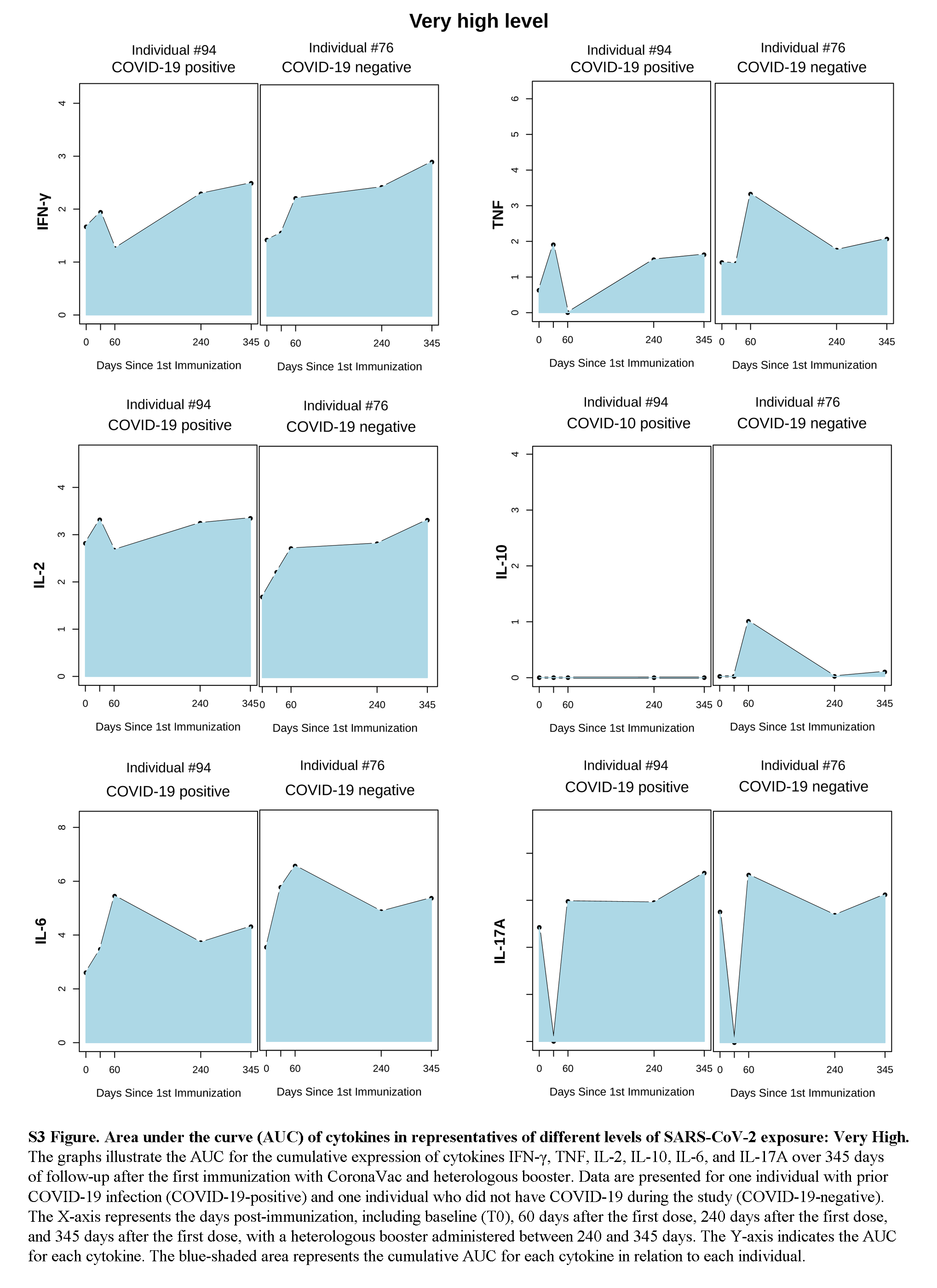

Supplement: Supplementary file 3 [file Image3.tif]

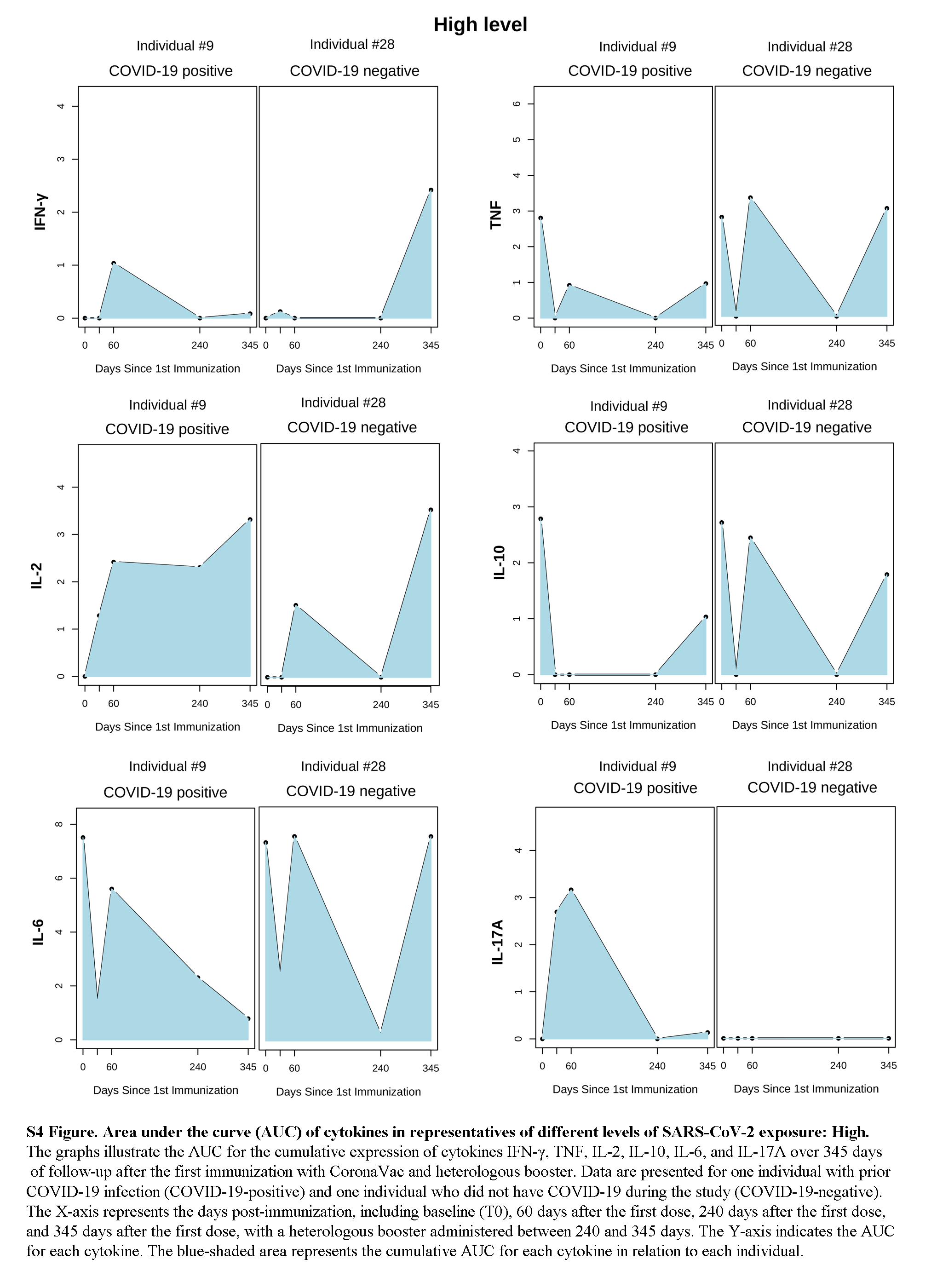

Supplement: Supplementary file 4 [file Image4.tif]

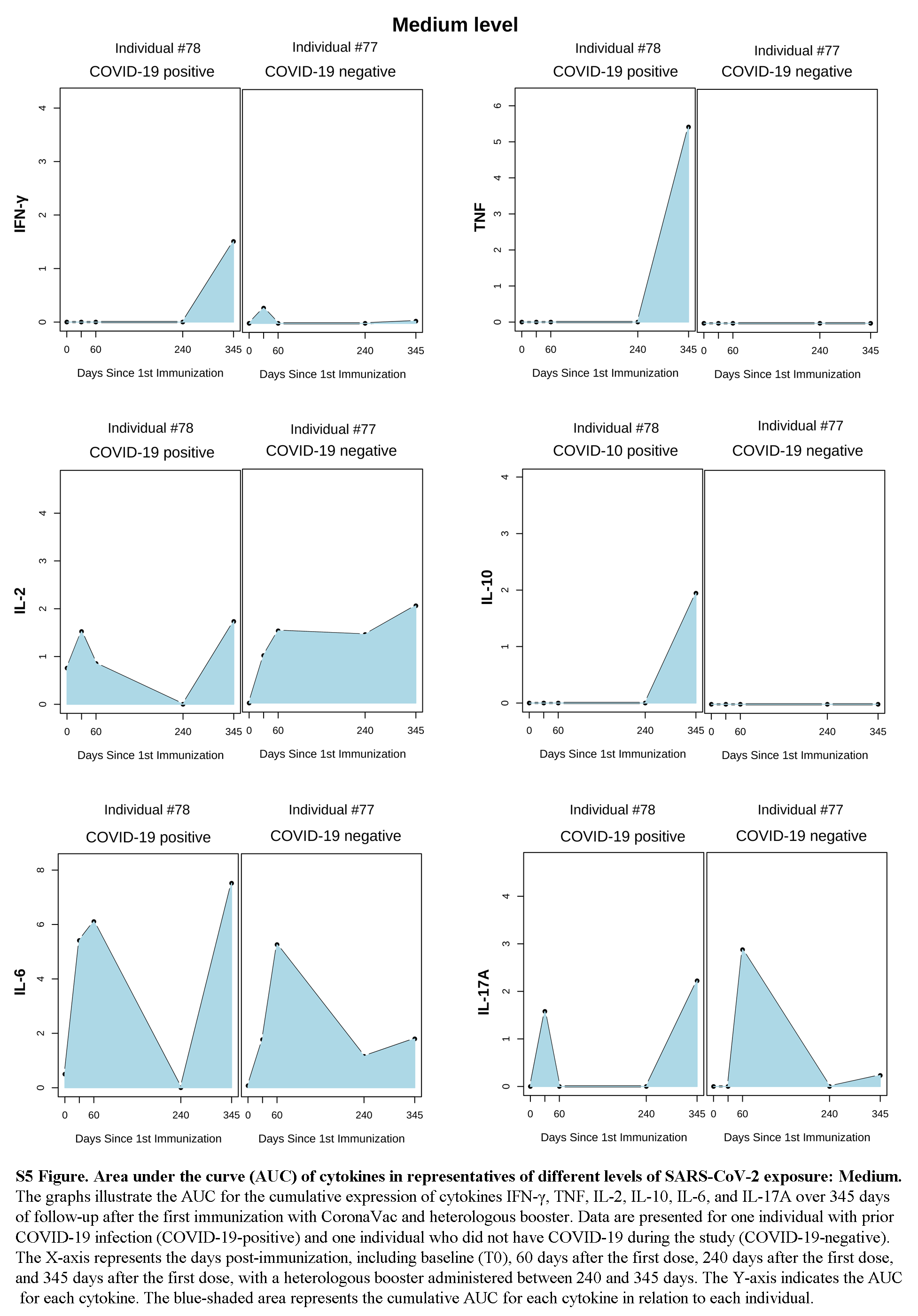

Supplement: Supplementary file 5 [file Image5.tif]

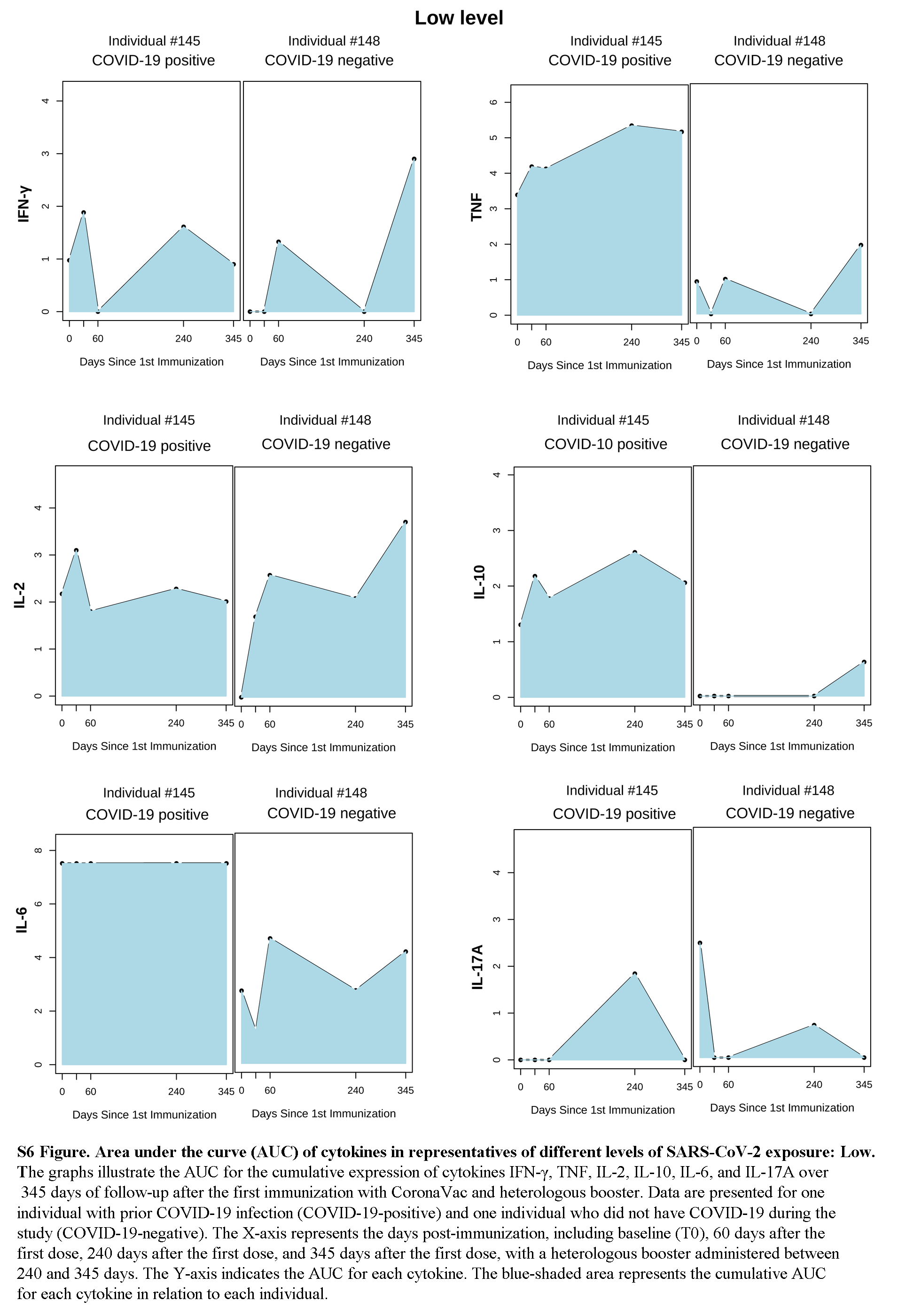

Supplement: Supplementary file 6 [file Image6.tif]

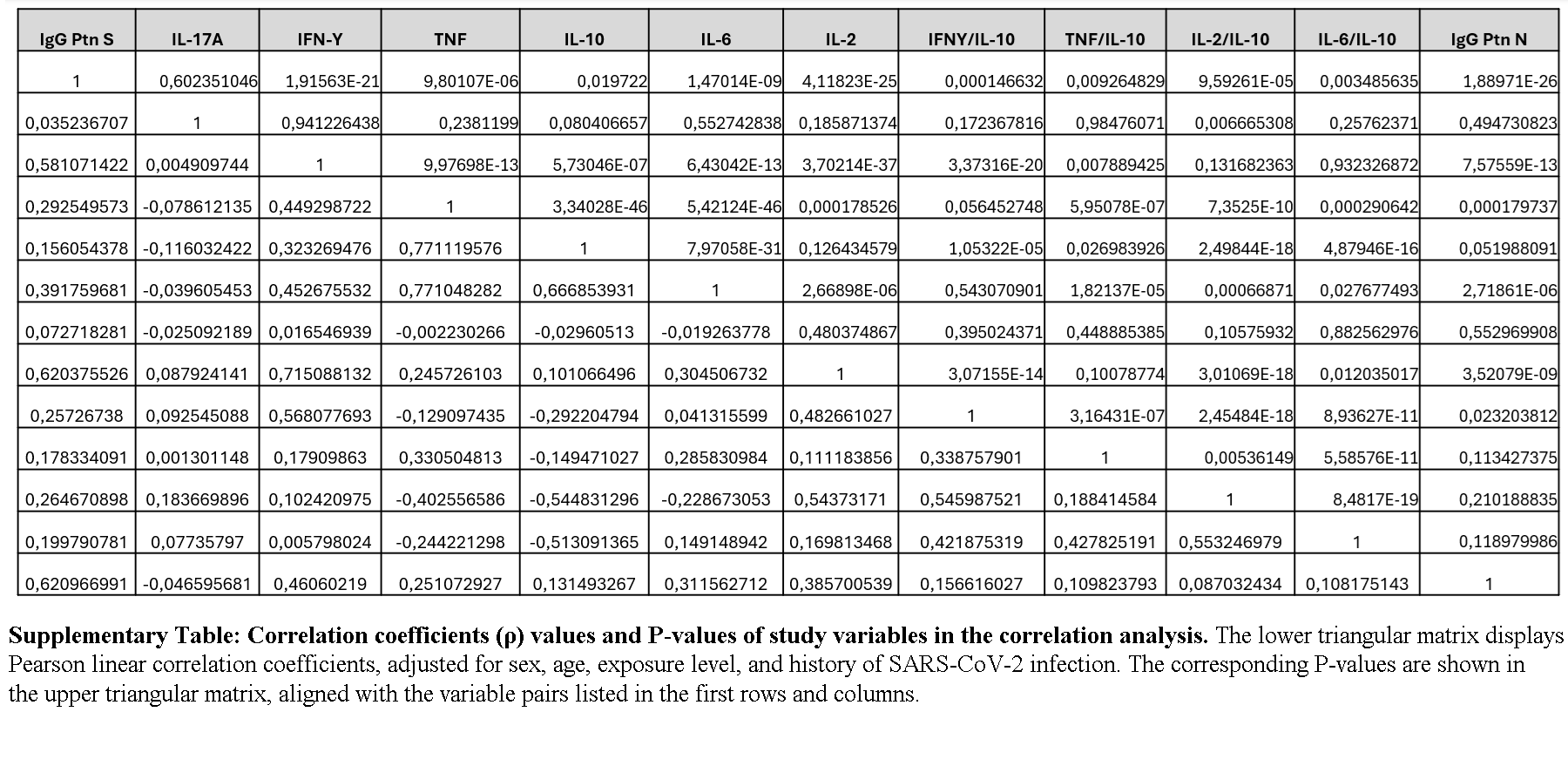

Supplement: Supplementary file 7 [file Image7.tif]
